# Conflict of interest and disclosure in healthcare: We can do better

**DOI:** 10.1002/acm2.14002

**Published:** 2023-04-23

**Authors:** Timothy D. Solberg

**Affiliations:** ^1^ Department of Radiation Oncology University of Washington Seattle Washington USA

The early part of my COVID experience coincided with a year in Washington, DC, working for the US Food and Drug Administration. The stresses within the FDA were significant, as were those surrounding the political landscape in DC at the time. Though I tried to focus my down time on activities intended to decrease anxiety levels, I nevertheless found myself transfixed on two miniseries, *Dopesick* and *The Dropout*, in part because each dealt with elements of healthcare and healthcare regulation that I could closely identify with. For this editorial, I will focus on *Dopesick* (Hulu, 2021), based on the 2018 bestseller by Beth Macy.^1^ As an aside, if you find the miniseries disturbing, you will find the book even more so.

The subject has been in the mainstream news for a number of years, so I think many of us are at least familiar with the general story—the manipulation by the pharmaceutical industry that led to the proliferation of opioid prescriptions and the resulting opioid epidemic in America. Pharmaceutical companies promoted “pain as fifth vital sign,” and the ten‐point pain scale with smiley faces and frowns that continues to be ubiquitous in doctors’ offices. Professional societies, which advocated for increased opioid use, received funding from the pharmaceutical industry.^2^ In 2012 alone, over 250 million prescriptions were written for opioids.^3^, ^4^ From 1999 to 2011, the use of prescription opioids, oxycontin, oxycodone, and hydrocodone, skyrocketed, as did deaths due to opioid‐related overdoses.^5^ Policy changes beginning at individual State levels began to reduce the availability of prescription opioids, resulting in a dramatic increase of the price on the illegal market, and a switch to lower‐cost alternatives of heroin and more recently, fentanyl. Overdose deaths have continued to increase year over year, with more than 106 000 in the U.S. in 2021.^6^

One highly successful tactic employed by the prescription opioid industry was the use of all‐expense‐paid “pain management” seminars targeting health care professionals and held at luxury resorts.^7^ The physician played by Michael Keaton in *Dopesick*, originally reluctant to prescribe opioids, attends such a seminar and meets with highly regarded physicians who reinforce the false claims promoted by the pharmaceutical industry. He is subsequently persuaded to speak at seminars as a pain expert himself. While we may cringe at the tactics in *Dopesick*, these sham educational programs, recruiting of medical specialists for speakers‐bureaus, and interactions with sales representatives continue to be highly effective marketing mechanisms. And while providers may truly believe that enticements do not alter their practice and prescribing patterns, there is ample evidence to the contrary.^7^, ^8^, ^9^

The Physician Payments Sunshine Act (the Sunshine Act), passed in 2010 as part of the Affordable Care Act, requires companies to report all financial relationships between physicians and the pharmaceutical and medical device industries for products covered by Medicare and Medicaid. The goal of the Sunshine Act was to increase financial transparency and to reveal potential conflicts of interest.^10^ Data collection began on 1 August 1 2013; the process is now supported the Open Payments system run by CMS (https://openpaymentsdata.cms.gov/) and includes a tool for searching the database.

In parallel, most reputable medical journals began to mandate disclosure of financial interest. Among prominent journals, the Journal of the American Medical Association states: “All authors are required to report potential conflicts of interest including specific financial interests relevant to the subject of their manuscript…” The International Journal of Radiation Oncology, Biology, Physics asks authors “… to disclose all relationships/activities/interests … that are related to the content of your manuscript.” Our own JACMP policy states: “Authors must disclose any financial interests, direct or indirect that might affect the conduct or reporting of the submitted work.”

Have either the Sunshine Act or the subsequent financial disclosure mandates made a difference? In a word, no. In 2019, using data from CMS Open, ProPublica reported that more than 2500 physicians had received at least half a million dollars apiece from the pharmaceutical and medical device industries in the prior 5 years.^11^ In the medical device industry, Rachal and Lim reported that in the 5 years since the Sunshine Act came into effect, contributions to doctors from the 20 top‐spending medical technology companies more than tripled.^12^, ^13^ In our own profession, Marshall et al. have reported that payments to radiation oncologists have actually increased since the Open Payments system was implemented and suggested that increased emphasis on financial conflicts of interest is needed.^14^ Lexchin and Fugh‐Berman performed an exhaustive review of the literature and concluded that “… there is no evidence that physician behavior regarding conflicts of interest has changed,” and that disclosure is “…not sufficient to address the damage that industry relationships causes to medical knowledge and public health.”^13^ Additionally, disclosure itself is highly variable. Within the medical device industry, several studies have shown COI reporting rates below 50%.^15^

And what about medical physicists? Well, payments to individuals are reported as a function of national provider identifier (NPI), so for better or worse, our profession is free from Open Payments requirements (as a caveat, some payments to institutions may be tagged with the name of an investigator). While one could make a valid argument that payments to medical physicists are negligible relative to health care as a whole, they nevertheless do occur. As I watched the various strategies employed by the industry and carried out by the pharmaceutical reps in *Dopesick*, a number of experiences in my own career began to hit home. Many of us, myself included, have accepted flights and lodging to attend seminars sponsored by manufacturers in the medical device industry. And many of us, again, myself included, have accepted fees to give talks on behalf of manufacturers in the medical device industry. Like many of the providers above, I'm sure we all believe we are being objective when we participate in these activities.

While a plane ticket, hotel room, modest speaker fee, or even a meal may seem trivial, such payments amount to billions of dollars every year, costs that get passed on to health care consumers. Additionally, the perverse incentives created can lead to wasteful health spending, with studies suggesting 25%−30% of overall health care spending could be considered waste.^16^ And as we witnessed from the opioid crisis, perverse incentives can have profound public health consequences.

So, perhaps it is time to reconsider how we handle payments and financial conflict of interest. Many institutions have prohibitions on vendors providing lunch or passing out pens, yet allow speakers’ and other personal fees that are orders‐of‐magnitudes greater. Let us end all fees paid to individuals!

We need to tighten the requirement on reporting. Many journals ask authors to report potential conflicts that could potentially be related to the work. As this allows individuals to define for themselves what is and is not a conflict, I would argue that any and all financial relationships be disclosed. And medical journals should more carefully vet manuscripts, authors, and reviewers for financial relationship.

Additionally, reporting financial conflicts of interest should be explicitly linked to terms of employment, particularly within academic health care institutions where publishing is an important criterion for advancement. Similarly, there should be explicit policies prohibiting involvement in purchasing decisions by individuals with related outside financial interests.

Professional organizations should insist on speakers with no financial conflicts of interest, and should not accept commercial support for educational events.^13^

Mechanisms for increased transparency for the healthcare consumer should be developed. As an example, the State of California recently passed AB 1278, requiring institutions and providers to provide patients with “…a written or electronic notice of the Open Payments database,” and to post an Open Payments database notice “…in each location where the licensee practices and in an area that is likely to be seen by all persons who enter the office.”^17^ A requirement similar to this should be nationally mandated.

The Open Payments reporting requirements should be extended to health professionals beyond those with a national provider identifier, and should include medical physicists as well as those in health care administration.

An outright ban on financial relationships would be unpopular, unenforceable a perhaps even counterproductive in some circumstances. Given the inescapable fact that financial relationships will always influence individual behavior, however, it is not reasonable to ask health professionals to voluntarily abstain from such relationships? Organizations could adopt something like a “health care financial relationships pledge” in place of or as part of an individual's COI disclosure. And provided the Open Payments system was extended to all health professionals, compliance with such a pledge could be easily verified. Just a thought.

## CONFLICT OF INTEREST STATEMENT

The author reports no relevant conflicts‐of‐interest related to this work. The author is a part‐time employee of the University of Washington, is a managing partner of Global Radiosurgery Services, and is the CEO and co‐founder of Foretell Medical. The author is a deputy editor‐in‐chief for the JACMP and is compensated by the AAPM for this service. No large language models were used in the preparation of this editorial.
